# Exposure to biodiesel exhaust is less harmful than exposure to mineral diesel exhaust on blood-brain barrier integrity in a murine model

**DOI:** 10.3389/fnins.2024.1440118

**Published:** 2024-09-13

**Authors:** Michael Nesbit, Colleen Kah Ling Ko, John C. L. Mamo, Virginie Lam, Katherine R. Landwehr, Alexander N. Larcombe, Ryu Takechi

**Affiliations:** ^1^Curtin Health Innovation Research Institute, Curtin University, Perth, WA, Australia; ^2^School of Population Health, Faculty of Health Sciences, Curtin University, Perth, WA, Australia; ^3^Curtin Medical School, Faculty of Health Sciences, Curtin University, Perth, WA, Australia; ^4^Respiratory Environmental Health, Wal-yan Respiratory Research Centre, Telethon Kids Institute, Perth Children’s Hospital, Perth, WA, Australia

**Keywords:** air pollution, diesel exhaust, biodiesel exhaust, blood-brain barrier, neuroinflammation

## Abstract

Emerging data suggest that air pollution is a persistent source of neuroinflammation, reactive oxygen species (ROS), and neuropathology that contributes to central nervous system (CNS) disorders. Previous research using animal models has shown that exposure to diesel exhaust causes considerable disruption of the blood-brain barrier (BBB), leading to marked neuroinflammation. However, the effects of biodiesel exhaust on cerebrovascular integrity and neuroinflammation have not been explored previously. Therefore, in this study, 8-week-old BALB/c mice were exposed to biodiesel exhaust (derived from canola biodiesel or tallow biodiesel) and compared with control mice that were exposed to air or mineral diesel exhaust. Consistently with previous findings, the integrity of the BBB was significantly disrupted by exposure to mineral diesel exhaust. Tallow and canola biodiesel exhaust exposure resulted in no BBB disruption. Moreover, both tallow and canola biodiesels significantly attenuated oxidative stress in the brain. The data collectively suggest that biodiesel exhaust may exert significantly less detrimental effects on brain function, compared to mineral diesel.

## Introduction

The blood-brain barrier (BBB) forms a protective barrier for the brain, insulating the central nervous system (CNS) from hazardous molecules in the blood. Dysfunction of BBB and subsequent parenchymal extravasation of neurotoxic molecules induces significant neuroinflammation and heightened oxidative stress (Takechi et al., 2017; Mamo et al., 2019). Such neuroinflammation and oxidative stress are commonly indicated in the aetiology of various neurodegenerative disorders (Block et al., 2007). For instance, in Alzheimer’s disease, BBB dysfunction and elevated neuroinflammation are established early neuropathophysiological hallmarks which precede neurodegeneration and cognitive impairment (Nation et al., 2019; Lam et al., 2021). However, the exact cause of BBB dysfunction in the pathogenesis of neurodegenerative disorders is not fully understood.

While multiple environmental factors have been identified to induce BBB disruption and neuroinflammation, epidemiological studies increasingly report that air pollution is one of the most prevalent causes of inflammation and oxidative stress, which potentially leads to the disruption of BBB (Craig et al., 2008). The combustion of fossil fuels such as mineral diesel is known to lead to substantial air pollution. Indeed, in our previous study, we reported that exposure to mineral (ultra-low sulfur diesel or ULSD) exhaust significantly disrupted BBB and caused neuroinflammation in otherwise healthy wild-type mice (Heidari Nejad et al., 2014). Other animal studies have also demonstrated that exposure to mineral diesel (371 ppm sulfur) exhaust causes significant pro-inflammatory responses as well as oxidative stress in the brain (Levesque et al., 2011).

Instead of such potentially harmful mineral diesel, there has been a growing need for a renewable fuel type compatible with most modern diesel engines. Amongst those, biodiesel, which is generated from renewable organic sources such as plant oils is one of the most well-developed and characterised. Despite the fact that biodiesel can directly replace mineral diesel in many engines (Graver et al., 2016), there are differences in physico-chemical composition between biodiesel exhaust and mineral diesel exhaust, and between different types of biodiesel depending on the feedstock used for creation (Landwehr et al., 2021; McCormick et al., 2003). Accumulating evidence indicates that combusting biodiesel instead of mineral diesel alters exhaust composition dependent on the fuel feedstock and may reduce the total *mass* of particulate matter, but lead to increases in the *number* of ultrafine particles and levels of toxic gases such as oxides of nitrogen (NO*_x_*) (Landwehr et al., 2021). However, there is considerable variability in results previously reported, primarily due to differences in measurement methodologies and the use of older-technology diesel engines (Landwehr et al., 2021). This means that it is currently very difficult to draw any firm conclusions regarding the relative toxicity of mineral diesel vs. different types of biodiesels. Our previous research, which has focussed on the respiratory system, indicates that there is a spectrum in toxic effects both *in vitro* (Landwehr et al., 2021) and *in vivo* (Landwehr et al., 2023), however there is a paucity of research which investigates the effects of biodiesel exhaust exposure on neurotoxicological outcomes, and in particular BBB and neuroinflammation. Valand et al. (2018) examined brain gene expression and histopathology in rats exposed to a variety of biodiesel blends for up to 28 days (6 h/day, 5 days/week). The overall finding was that B7 (the biodiesel blend containing the greatest proportion of mineral diesel) exposure typically led to more differently expressed genes associated with antioxidant defences and inflammation compared with blends containing more biodiesel. Similarly, using an indirect model of exposure of rat primary cortical cells, Gerber et al. (2024) showed that exhaust particles derived from B7 rapeseed methyl ester fuel led to higher neurotoxic potency than exposure to particles derived from B50 rapeseed methyl ester (Valand et al., 2018). Collected particles were used, so the potential contribution of exhaust gasses is unknown. The authors attribute this greater potency to chemicals adsorbed onto the particles, as exposure to pure carbon particles using the same model, led to very limited effects.

In response to this lack of knowledge, the current study was designed to examine the effects of a single exposure and multiple (eight) exposures to biodiesel exhaust, derived from canola and tallow, on BBB integrity and neuroinflammation in healthy wild-type mice. We selected canola and tallow-derived biodiesel because they are commonly used worldwide and because they resulted in the most extreme outcomes in our previous *in vitro* testing (Landwehr et al., 2021). In the present study, we also compared the effects with mineral diesel exhaust. Based on our previous findings, we hypothesized that exposure to tallow biodiesel exhaust would result in more severe neurotoxicological outcomes compared with mineral diesel exhaust exposure, while exposure to canola biodiesel exhaust would be the least harmful.

## Materials and methods

### Animals

Murine experiments described here were approved by Curtin University Animal Ethics Committee accredited by the National Health and Medical Research Council (NHMRC) (Approval No. ARE2020-16). Eight-week-old male BALB/c mice were supplied by Animal Resources Centre (Murdoch, WA, Australia). The mice were acclimated to the housing facility for a week, on a 12 h light/dark cycle. Standard “Rat and Mouse Cubes” chow (Specialty Feeds, Glen Forrest, WA, Australia) and water were available *ad libitum*. We used 12 mice per group.

### Exhaust exposure

The mice were randomly assigned to either control (air), mineral diesel (ULSD), tallow biodiesel exhaust (“tallow”), or canola biodiesel exhaust (“canola”) groups (*n* = 12 per group). The mice were exposed to exhaust generated by a diesel engine as per our method published previously, which includes the presentation of key components of each exhaust (Landwehr et al., 2021, 2023). Briefly, the mice were loaded into an exposure chamber (a 35 cm × 35 cm × 35 cm plastic box with internal dividers separating individual mice) and exposed to diluted exhaust generated from a light/medium diesel engine under constant 40% load and run at 2,000 rpm for 2 h once, or 2 h per day for eight consecutive days. The exhaust gases and particles exiting the engine travelled into a dilution chamber, which was diluted 1/10 with filtered ambient air with cold start emissions included as part of the exposure. The control group mice were exposed only to filtered air.

### Brain tissue collection

Twenty-four hours after the last exposure, mice were assessed for respiratory outcomes as previously reported (Landwehr et al., 2023) prior to euthanasia. As a preparation for immunofluorescence microscopy, brain tissues were carefully removed, rinsed in phosphate buffered saline (PBS), flash-frozen in liquid nitrogen and stored at −80°C.

### Assessment of BBB integrity

As previously described, parenchymal extravasation of IgG assessed with immunofluorescent microscopy was used as a marker of BBB disruption (Elahy et al., 2015). Briefly, 20 μm brain cryosections were fixed in 4% paraformaldehyde in 0.1 M phosphate buffer at room temperature, washed 3× in PBS and incubated in 10% goat serum in PBS to block unspecific binding sites.

Goat anti-laminin a4 antibody (1:200, R&D) was incubated at 4°C for 20 h in Antibody Signal Enhancing Buffer (ASE) (Rosas-Arellano et al., 2016). Sections were washed 3× in PBS and incubated with donkey anti-goat 555 (1:500, Thermo A32816) for 3 h at RT. Sections were washed in PBS again and incubated with goat anti-mouse IgG 488 (1:200, Thermo).

The nuclei were counterstained with Hoechst 33342 (1:500, Thermo) before being mounted with antifade mounting media. Immunofluorescent micrographs were captured with a slide scanner AxioScan Z1 (Zeiss, Oberkochen, Germany) using a 20× 0.8NA objective. Four Z-stacks at intervals of 2 μm were acquired and automatically combined using the Extended Depth of Focus “Wavelets” algorithm in Carl Zeiss ZEN Blue 3.1. The pixel intensity of fluorescence signals of the parenchymal extravasated IgG in the peri-vascular area was quantitatively measured within the regions of cortex and hippocampal formation as previously published (Nesbit et al., 2021).

### Evaluation of oxidative stress and neuroinflammation

The expression of 8-hydroxyguanosine (8OHG) and glial fibrillar acidic protein (GFAP) in the cortex and hippocampus were used as markers for oxidative stress and astrogliosis, respectively by using previously established techniques (Takechi et al., 2017; Mamo et al., 2019). The brain cryosections were incubated for 20 h at 4°C with a mixture of mouse anti-8OHG (1:500, Abcam) and goat anti GFAP (1:500, Abcam) in ASE. Slides were washed and subsequently incubated with donkey anti-goat Alexa647 (1:500) for 2 h in PBS, washed, then incubated with goat anti-mouse Alexa488 (1:500). As described in the previous section, the immunofluorescent micrographs were captured with Zeiss AxioScan Z1 and the pixel intensity of 8OHG and GFAP staining was quantitatively measured within the cortex and hippocampus using Intellesis Trainable Segmentation.

### Statistical analysis

Based on our previous study reporting the effects of diesel exhaust on BBB, each experimental group comprised of 12 mice, which was expected to produce sufficient statistical power (Heidari Nejad et al., 2014). Following the data normality test using D’Agostino Spearman test, two-way ANOVA with Fisher’s LSD multiple comparison was used to determine the effects of biodiesel exhaust exposure on BBB integrity and neuroinflammation (GraphPad Prism 9). Statistical significance was assessed at *p* < 0.05.

## Results

### In contrast to mineral diesel exhaust, exposure to biodiesel exhaust did not induce BBB dysfunction

**Figure 1:**
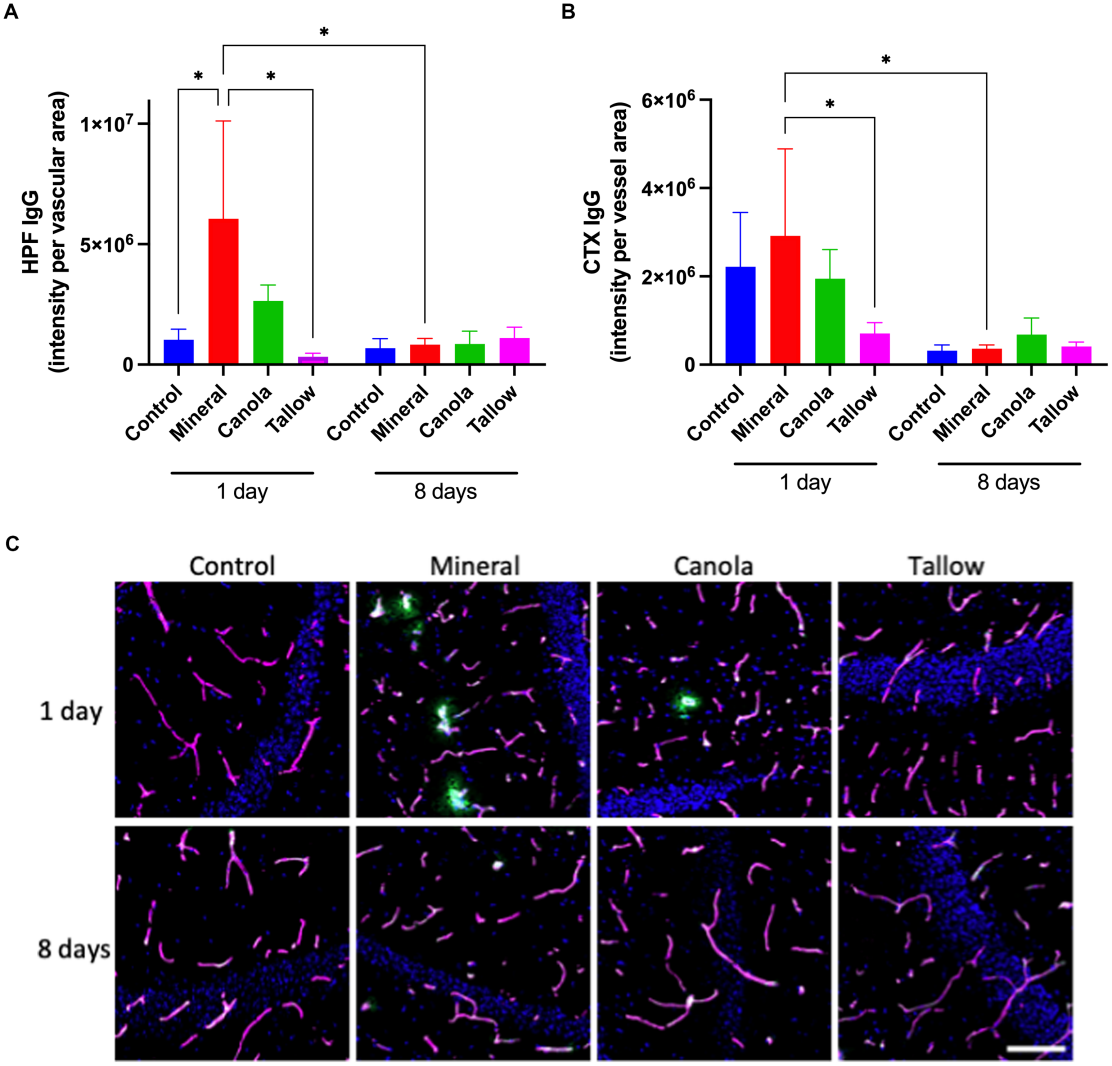
Blood-brain barrier integrity. The integrity of blood-brain barrier (BBB) was assessed by measuring the parenchymal peri-vascular extravasation of IgG with immunofluorescent microscopy in mice that were exposed to either air (control), mineral diesel, canola, or tallow biodiesel exhaust for 2 h/day for 1 day, or for 2 h/day for 8 consecutive days. **(A)** The pixel intensity of IgG extravasation in the hippocampal formation (HPF) is presented as per vascular area measured with laminin-a4 staining. **(B)** The pixel intensity of IgG extravasation of cortex (CTX) is presented. **(C)** The immunofluorescent micrographs are representative images of IgG extravasation (green) with laminin-a4 (magenta) showing vascular area of HPF. Nuclei were stained with DAPI (blue). The scale bar indicates 50 μm. Statistical significance was assessed with two-way ANOVA followed by Fisher’s LSD *post hoc* test. Statistical significance is expressed with * at *p* < 0.05, *F* = 1.541 (HPF) and 1.756 (CTX), *n* = 5–8.

Consistent with our previous study, a single exposure to mineral diesel exhaust in wild-type mice induced a significant increase in hippocampal peri-vascular extravasation of IgG compared with control mice exposed to air (*p* = 0.0312), indicating the breakdown of BBB (Figure 1A). The hippocampal IgG extravasation in canola mice was approximately 40% of that of mineral diesel, although it was not significantly different from the mineral or control mice (*p* = 0.079). Conversely, exposure to tallow biodiesel exhaust for 2 h resulted in a significant reduction of hippocampal IgG extravasation compared with mineral diesel.

In the cortex, a single exposure to mineral diesel exhaust did not result in a significant increase in parenchymal IgG compared with control (Figure 1B). Nonetheless, exposure to tallow biodiesel exhaust led to a significant reduction of IgG extravasation, compared with the mineral diesel group mice (*p* = 0.0492). IgG extravasation in the cortex of canola mice also showed a modest reduction compared to the mineral group and was comparable to control.

Exposure to mineral diesel exhaust for 8 consecutive days resulted in a significant attenuation of IgG extravasation both in hippocampus (*p* = 0.0251) and cortex (*p* = 0.0361), compared with the single exposure, and showed no significant increase compared to control (Figure 1). The IgG extravasation levels in mice exposed to canola or tallow biodiesel exhaust for 8 days were comparable to controls and mineral diesel mice.

### Repeated biodiesel exposures indicate significantly less oxidative stress

**Figure 2:**
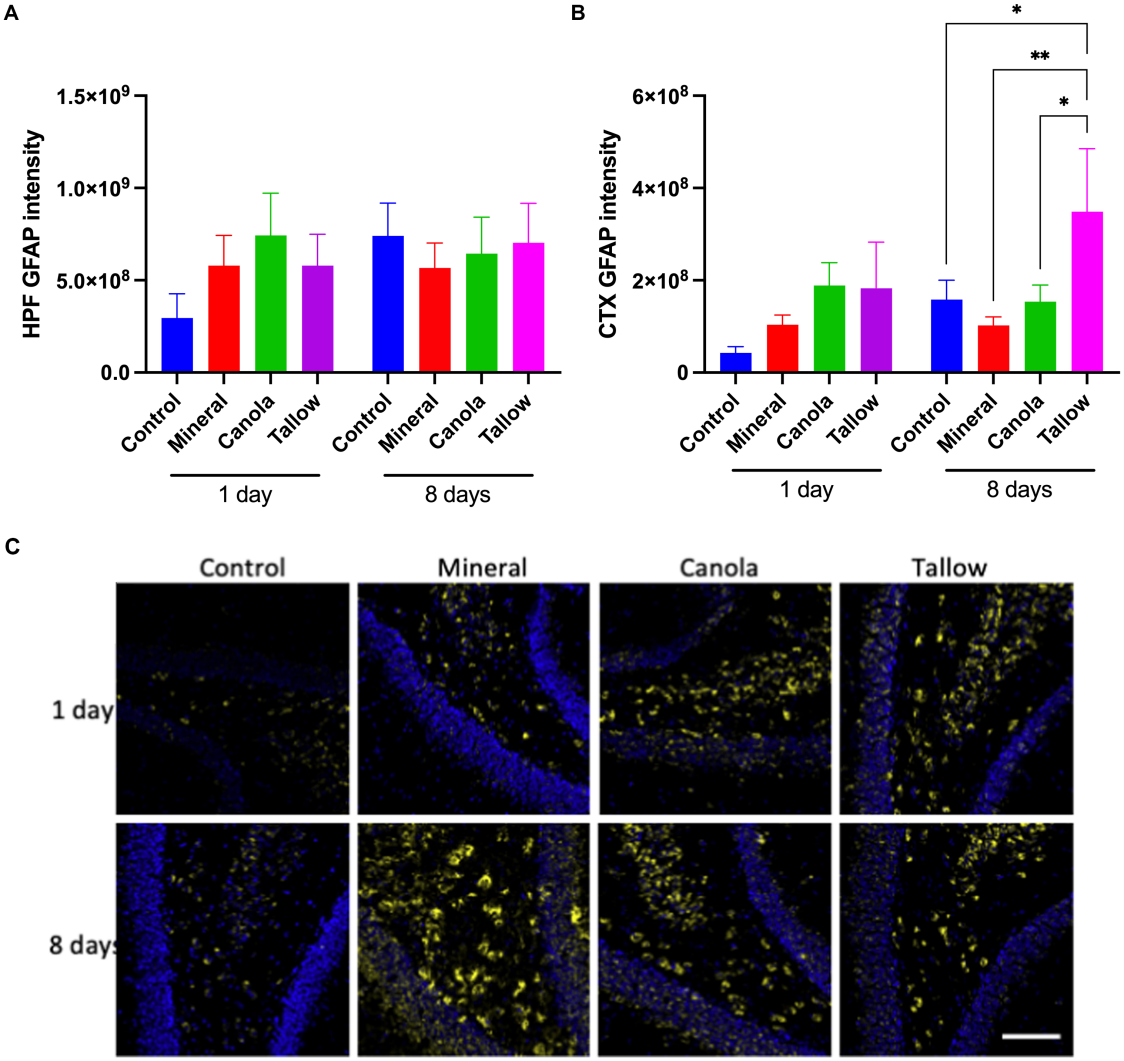
Oxidative stress. The levels of oxidative stress after exposure to either air (control), mineral diesel, canola, or tallow biodiesel exhaust for 2 h/day for 1 day, or for 2h/day for 8 consecutive days was assessed by measuring the parenchymal abundance of DNA damage marker, 8-hydroxyguanosine (8OHG) with immunofluorescent microscopy. The pixel intensity of 8OHG is presented as per the area in the hippocampal formation (HPF) **(A)** and cortex (CTX) **(B)**. **(C)** The representative immunofluorescent micrographs showing 8OHG in yellow in HPF and nuclei in blue are presented. The scale bar indicates 100 μm. Statistical significance was assessed with two-way ANOVA followed by Fisher’s LSD *post hoc* test. Statistical significance is expressed with * at *p* < 0.05 and ** at *p* < 0.01, *F* = 1.885 (HPF) and 1.554 (CTX), *n* = 5–9.

There was no significant effect of a single 2 h exposure to mineral diesel exhaust on 8OHG of the hippocampal formation or cortex, indicating a comparable level of oxidative stress compared with control (Figure 2). Exposure to canola and tallow biodiesel exhaust showed increasing trends in hippocampal and cortical 8OHG compared to the control and mineral diesel groups, although these were not statistically significant.

Eight consecutive days of 2 h exposures to mineral diesel exhaust resulted in a significant ~5-fold increase in 8OHG in both hippocampus (*p* = 0.0037) and cortex (*p* = 0.0172), compared to a single 2 h exposure. However, these increases were not significantly higher compared with control mice that had 8 days of exposures to air. 8OHG expression in the hippocampal formation was significantly (~80%) lower in canola (*p* = 0.207) and tallow (*p* = 0.0122) exhaust exposed mice compared with mineral diesel.

### Biodiesel exhaust increases astrocytic GFAP

**Figure 3:**
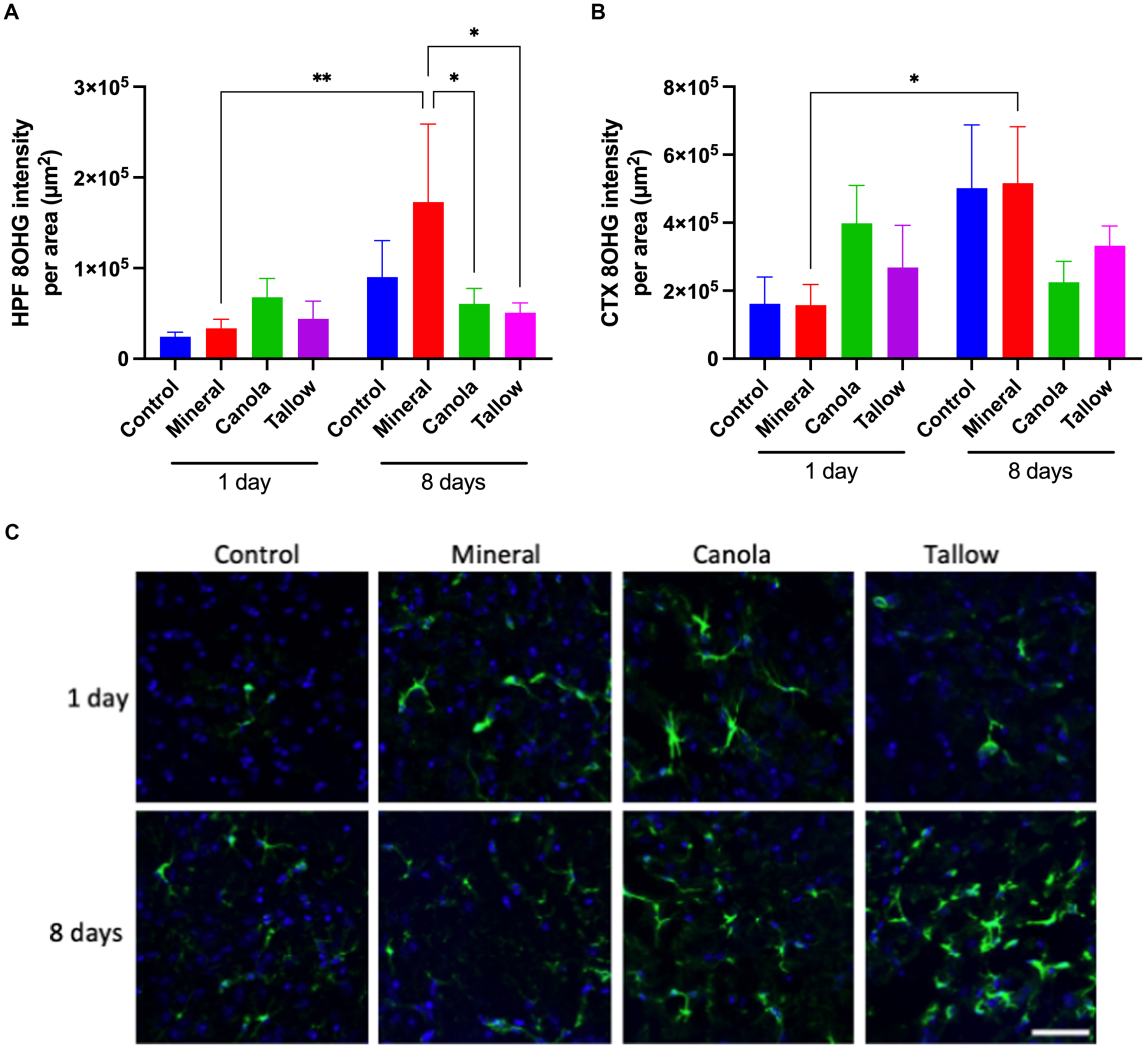
Cerebral GFAP expression. The abundance of glial fibrillary acidic protein (GFAP) was used to assess the levels of astrocyte activation as a marker of neuronal damage and inflammation in mice exposed to either air (control), mineral diesel, canola, or tallow biodiesel exhaust for 2 h/day for 1 day, or 2 h/day for 8 consecutive days. **(A)** The pixel intensity of GFAP is presented as per the area in the hippocampal formation (HPF) **(A)** and cortex (CTX) **(B)**. **(C)** The representative immunofluorescent micrographs showing GFAP in green in CTX and nuclei in blue are presented. The scale bar indicates 50 μm. Statistical significance was assessed with two-way ANOVA followed by Fisher’s LSD *post hoc* test. Statistical significance is expressed with * at *p* < 0.05 and ** at *p* < 0.01, *F* = 0.5070 (HPF) and 1.999 (CTX), *n* = 5–9.

Mice exposed to mineral diesel exhaust for 2 h showed no statistical differences in hippocampal and cortical expression of GFAP compared to air exposed controls (Figure 3). Both canola and tallow biodiesel groups also showed no significant changes to the hippocampal and cortical GFAP levels after a single exposure. Similarly, exposure to mineral diesel or canola biodiesel exhaust for 2 h per day for 8 consecutive days led to no significant alterations in GFAP expression in the hippocampal formation and cortex (Figure 3). Mice exposed to tallow biodiesel exhaust for 2 h per day for 8 days showed a significant increase in cortical GFAP expression, compared to control (*p* = 0.0375), mineral diesel (*p* = 0.0080), and canola diesel (*p* = 0.0395) groups.

## Discussion

Exposure to mineral diesel exhaust has previously been shown to result in significant BBB dysfunction, leading to modestly heightened neuroinflammation (Heidari Nejad et al., 2014; Ehsanifar et al., 2021). The current study was the first to test the effects of exhaust generated by the combustion of biodiesel fuel on BBB. We confirmed that a single exposure to mineral diesel exhaust for 2 h induces significant BBB disruption in healthy wild-type mice, leading to cerebral parenchymal extravasation of IgG, a finding consistent with our previous report (Heidari Nejad et al., 2014). Despite the substantial disruption of BBB, a single exposure to mineral exhaust did not result in significant increases in cortical and hippocampal GFAP or 8OHG, suggesting no effects on astrocyte activation or oxidative stress after a single exposure.

Our results showed that multiple exposures to mineral diesel exhaust over 8 days normalised BBB permeability, which may be due to adaptation of BBB endothelium to the repeated insults. Similar endothelial adaptation has previously been reported, whereby Uzarski et al. (2013) demonstrated that endothelial cells that were exposed to variable shear stress showed marked adaptation in gene and cytokine expression, leading to improved endothelial functionality. Although, it needs to be noted that the brain samples were collected 24 h after the exposure to air/diesel exhaust, and BBB damage might have been evident in earlier timeframe. Nevertheless, repeated exposure to mineral diesel exhaust resulted in a significantly elevated hippocampal and cortical 8OHG, compared to the mice exposed to mineral diesel exhaust for just 2 h.

8OHG is an RNA nucleoside which is known as the oxidative derivative of guanosine (Bello et al., 2022). Thus, 8OHG level was employed as a marker of oxidative stress that causes DNA damage. Nano-sized particles such as the particulate matter found in mineral diesel exhaust are reported to pass the BBB and physically penetrate the CNS of animals in modest quantities (Calderon-Garciduenas and Ayala, 2022), which may explain the elevation of oxidative stress in the absence of significant BBB breakdown in mice that were exposed to mineral diesel for 8 days.

In contrast to the finding that mineral exhaust exposure significantly induced BBB disruption, our data showed that single exposure to biodiesel exhaust from canola and tallow biodiesel had no detrimental effects on BBB integrity. Particularly, the hippocampal and cortical parenchymal IgG extravasation was significantly lower in tallow biodiesel exposed mice, compared with mineral exhaust mice. After the 8-day exposure to canola or tallow biodiesel exhaust, BBB integrity was comparable to the control mice exposed to air. Whilst we did not investigate the underlying mechanisms, a number of studies report that oxides of nitrogen (NO*_x_*) such as nitric oxide (NO) lead to an increase in BBB permeability due to an elevated production of peroxynitrite (Chen et al., 2018).

In line with the lack of detrimental effects of biodiesel exhaust on BBB integrity, oxidative stress after the 8-day exposure was significantly attenuated in both canola and tallow biodiesel exhaust exposed mice, compared to the mineral diesel exhaust exposed mice. Similarly, the single or repeated exposures to biodiesel exhaust did not result in elevation in cerebral GFAP expression, except for the significantly increased cortical GFAP observed after the 8-day exposure to tallow biodiesel exhaust. A surge in astrocytic activation has been observed in brains of adult and neonatal mice following acute and sub-chronic intranasal or inhalation exposure with PM_2.5_ (particles with diameters smaller than 2.5 μm) (Gomez-Budia et al., 2020). In contrast, an emerging number of studies indicate that the increase of GFAP and activation of astrocytes are the implication of defence mechanisms and are rather beneficial. For example, it has been demonstrated in traumatic brain injury, astrocytes play central role in repairing BBB (Zhou et al., 2020). Furthermore, in stroke, it is reported that astrocytes promote angiogenesis and neurogenesis, supporting nerve recovery (Zhang et al., 2021). Since the brain samples were collected 24 h after the exposure to diesel exhaust, biodiesels may have no detrimental effects on the latter “recovery” process, upregulating GFAP and attenuating 8OHG, whilst this protection was disrupted in mice exposed to mineral diesel exhaust. Further investigation is needed to explore the mechanism for the transient increase in GFAP induced by a repeated exposure to tallow biodiesel exhaust.

One limitation of the study was that the tissue samples were collected 24 h after the last exhaust exposure, and thus, we were unable to assess the immediate response of BBB permeability and neuroinflammation. Future studies may consider multiple time points to assess these measures following the diesel exhaust exposure. In addition, we only used parenchymal IgG activation as a marker of BBB dysfunction, as per our published protocol. Future studies may include additional measures such as the expression of endothelial tight junction proteins to strengthen the findings.

The overall findings of this study suggest that short-term exposure to tallow or canola biodiesel exhaust has significantly less detrimental effects on BBB and neuronal inflammation and oxidative stress compared to similar exposure to mineral diesel exhaust. These observations collectively imply that, in this particular model, these two types of biodiesels exert significantly lesser detrimental effects on the brain compared with mineral diesel exhaust and may, by extension, reduce the risks of some neurological disorders.

## Data Availability

The original contributions presented in the study are included in the article/supplementary material, further inquiries can be directed to the corresponding author.
